# Vitamin A Promotes Leydig Cell Differentiation via Alcohol Dehydrogenase 1

**DOI:** 10.3389/fendo.2018.00644

**Published:** 2018-10-29

**Authors:** Yan Yang, Jiao Luo, Dan Yu, Tiantian Zhang, Qilian Lin, Quan Li, Xupeng Wu, Zhijian Su, Qihao Zhang, Qi Xiang, Yadong Huang

**Affiliations:** ^1^Guangdong Provincial Key Laboratory of Bioengineering Medicine, Department of Cell Biology, Jinan University, Guangzhou, China; ^2^Department of Biomedical Engineering, Jinan University, Guangzhou, China; ^3^Department of Pharmacology, Jinan University, Guangzhou, China

**Keywords:** vitamin A, Leydig cell, differentiation, alcohol dehydrogenase 1, retinoic acid, steroidogenic factor 1

## Abstract

Vitamin A (retinol) is important for multiple functions in mammals. In testis, the role of vitamin A in the regulation of testicular functions is clearly involved in rodents. It is essential for sperm production. Vitamin A deficiency adversely affects testosterone secretion. Adult Leydig cells are responsible for testosterone production in male. The role of vitamin A in regulating the differentiation of Leydig cells is still unknown. In this study, we explored the roles and underlying mechanisms of vitamin A in Leydig cell differentiation. We found that vitamin A could regulate the Leydig cells differentiation. Leydig cell differentiation is adversely affected in mice maintained on a vitamin A-free diet. This effect is mediated by alcohol dehydrogenase 1 (ADH1). ADH1 could increase retinoic acid (RA) synthesis, then RA facilitates Leydig cell differentiation by activating the steroidogenic factor 1 gene (*Nr5a1*) promoter activity, which consequently promotes Leydig cell specific gene expression, resulting in progenitor Leydig cells differentiation into functional Leydig cells. This is the first study connecting a metabolic enzyme of retinol (ADH1) to the the regulation of Leydig cell differentiation, which will provide experimental evidence for the development of therapeutics to promote Leydig regeneration through the administration of a RA signaling regulator or a vitamin A supplement.

## Introduction

Vitamin A is a group of unsaturated nutritional organic compounds. The common type of vitamin A includes retinol, retinal, retinoic acid, and several provitamin A carotenoids (most notable beta-carotene) (1). Vertebrates cannot synthesize vitamin A, which is generally acquired from plants and food (2). Vitamin A is usually transported via serum as retinol (ROL) bound to retinoid-binding proteins, and conversion of this retinoid for either storage (retinyl esters) or use (retinoic acid, RA) in target tissues (3–5). RA regulates many events during vertebrate development and controls many aspects of cell proliferation, differentiation, and apoptosis, reproduction, vision, and immune system (6–8). RA is produced by two steps enzymatic process from dietary vitamin A. The oxidation of retinol to retinaldehyde is controlled by the alcohol/retinol dehydrogenases (ADHs or RDHs) and by the short-chain dehydrogenases (9, 10). The conversion of retinaldehyde to RA is regulated by the aldehyde dehydrogenases (ALDHs) (11–13). Distinct cell type express particular enzymes.

In testis, vitamin A (retinol) and its principal biologically active derivative (RA) are clearly involved in the regulation of testicular functions in rodents (14). And the role of dietary vitamin A/retinol in normal spermatogenesis has been recognized for decades (15). Spermatogenesis can be inhibited by removing vitamin A from the diet of a mouse, and spermatogenesis can be reinitiated with vitamin A replacement. Vitamin A deficiency adversely affects testosterone secretion (14, 16). Adult Leydig cells are response for testosterone production (17). The role of vitamin A in regulation of Leydig cells differentiation is still unknown. And its mechanism of action in Leydig cells differentiation has not been elucidated.

Leydig cells, also known as interstitial cells of Leydig, distribute in clusters between the seminiferous tubules in the testicle and are the primary source of androgen in the male body (18). The differentiation of Leydig cell is an important process, in which Leydig precursor cells differentiate into functional mature Leydig cells. Steroidogenic factor 1 (SF1, NR5A1) plays important roles throughout the development of gonads and Leydig cell (19). *Nr5a1*-deficient mice also exhibit defects in gonadotrope gene expression and function (20). To better define the role of *Nr5a1*, specifically in Leydig cells, the Cre-Lox approach was used to generate a Leydig cell-specific *Nr5a1* knockout. These Leydig cell-specific *Nr5a1*-deficient mice have female external genitalia, are sterile, and exhibit no post-natal sexual maturation (21). Consequently, several steroidogenic enzyme-encoding genes, including steroidogenic acute regulatory protein (*Star*), P450 side-chain cleavage enzyme (*Cyp11a1*), 3b-hydroxysteroid dehydrogenase (*Hsd3b*), 17a-hydroxylase (*Cyp17a1*), and 17b-hydroxysteroid dehydrogenase 3 (*Hsd17b3*), which are known to be regulated by Nr5a1, are not expressed (22, 23). Additionally, it has been reported that Nr5a1 could induce murine mesenchymal stem cells (24), marrow stromal cells (MSCs) (25) or pluripotent stem cells into testosterone-producing Leydig cells (26). In our previous study, we also demonstrated embryonic stem cells (ESCs) could be induced to differentiate into Leydig cells by a combination of Nr5a1 with small molecule compounds (27). Together, these data suggested that *Nr5a1* plays a crucial role in Leydig cell differentiation.

Therefore, in this study, the roles of vitamin A in Leydig cell differentiation are determined. Meanwhile, its mechanism of action in Leydig cell differentiation will be studied and revealed, so as to provide a better understanding of the interaction and offer clearer explanations for the vitamin A and Leydig cell differentiation.

## Materials and methods

### Animals and treatments

C57BL/6 mice and Sprague-Dawley rats (at 8 weeks of age) from the experimental animal center of Guangdong Province were kept under conditions with controlled temperature (24 ± 1°C), relative humidity (50–60%), and a light/dark cycle of 12/12 h with standard rodent diet and drinking water. The experimental procedures were approved by the Institutional Animal Care and Use Committee of Jinan University. Weanling mice were kept with vitamin A-free diet (completely devoid of vitamin A, purchased fromTrophic Animal Feed High-tech Co., Ltd, JiangSu, China) for 90 days. The control mice were fed with regular diet and analyzed the same day. Male Sprague-Dawley rats were administered a single intraperitoneal (i.p.) injection of ethylene dimethanesulfonate (EDS, an alkylating toxicant that sellectively eliminates adult Leydig cell) synthesized as previously described (28) and dissolved in DMSO (Sigma-Aldrich, Poole, Dorset, UK) at a dose of 75 mg/kg body weight) on day 1, and 4-methylpyrazole (4-MP, Sigma, Poole, Dorset, UK) was injected i.p. every day during days 7–35 after EDS treatment. Testes from all animals were removed at 7 and 35 days after EDS treatment. Subsequently, the testes were decapsulated and incubated with 0.25 mg/mL collagenase D (Roche Molecular Systems, CA, US) in DMEM (Thermo Fisher Scientific, Waltham, MA, USA) in a shaking water bath (120 cycles/min) at 37°C for 15 min. After incubation, cold DMEM was added to stop the action of collagenase D. Seminiferous tubules were separated from the interstitial cells by gravity sedimentation. The cells were collected by centrifugation (300 g for 6 min) and washed with cold phosphate-buffered saline (PBS) and the cell pellet resuspended in radioimmunoassay precipitation assay buffer (RIPA). Lysates were centrifugated at 10,000 g for 20 min and protein concentration of the cleared lysate was determined.

### Isolation of progenitor leydig cells (PLCs) and adult leydig cells (ALCs)

To isolate progenitor and adult Leydig cells, 20 mice (21 days postnatal) and 10 mice (56 days postnatal) were used, respectively. The testes were incubated with 0.25 mg/mL collagenase D (Roche Molecular Systems, CA, US) in DMED for 10 min at 34°C. The dispersed cells were filtered through two layers of 100 mm-pore-size nylon mesh, centrifuged at 250 g for 10 min and resuspended in 55% isotonic Percoll to separate the cells based on their buoyant density. And centrifuged at 23,500 g and 4°C for 45 min, the fractions of progenitor Leydig cells with densities between 1.068 and 1.070 g/mL, and adult Leydig cells with densities of 1.070 g/mL were collected. The cells were cultured at 34°C for 24 h.

### Stable transfection of SF-1 mouse ESCs (mESCs-SF1)

Stable transfection of SF-1 mouse ESCs was conducted as we described previously (27). In brief, mouse Sf-1 cDNA was amplified from the testis by reverse transcription–polymerase chain reaction (RT-PCR), using forward primer 5′-ACTGAATTCGATATGGACTATTCGTACGACGAGGACCTGG-3′ and reverse primer 5′-TTAGGATCCTCAAGTCTGCTTGGCCTGCAGCATCTCAATGA-3′, cloned into the lentiviral pLVX-EF1a-IRES-ZsGreen1 Vector (Clonetech), and confirmed by sequencing. SF-1 lentiviral particles were packaged into NIH 293T cells following the manufacturer's protocol. For stable transfection, ESCs were infected with Sf-1 lentiviral particles overnight, and subsequent green fluorescence protein (GFP) gene expression was monitored by fluorescence microscopy and flow cytometry.

### Differentiation of SF1-overexpressing mESCs toward leydig cells

SF1-overexpressing mouse ESCs (mESCs-SF1) were cultured on mouse embryonic fibroblasts (MEFs) feeder treated by mitomycin-C in Knockout™ Dulbecco's Modified Eagle's Medium (DMEM; Thermo Fisher Scientific, Waltham, MA, USA), supplemented with 15% Knockout™ Serum Replacement (KSR; Thermo Fisher Scientific, Waltham, MA, USA), 2 mM Gluta MAX™-I (Thermo Fisher Scientific, Waltham, MA, USA), 1% nonessential amino acids, 0.1 mM 2-mercaptoethanol, 1% penicillin–streptomycin, and 1,000 U/mL leukemia inhibitory factor (LIF, Millipore, Darmstadt, Germany), and the culture medium was changed daily. Adherent SF1-overexpressing mESCs were dissociated using the StemPro accutase cell dissociation reagent (Thermo Fisher Scientific, Waltham, MA, USA). Embryoid body (EB) was formed by a hanging drop technique (800 cells in 20 μl of culture medium without LIF). After 5 days of culture, EBs were plated on gelatin-coated dishes and cultured in DMEM supplemented with 10% FBS, 8-Br-cAMP (Sigma, Poole, Dorset, UK) and forskolin (FSK; Sigma, Poole, Dorset, UK) (27). To study whether ADH1 contribute to Leydig cells differentiation. mESCs-SF1 cells were grown in Leydig cell differentiation (LC DM) supplemented with 1.5 mM 4-MP or LC DM with the 4-MP and 2.5 μM RA signaling agonist all-trans retinoic acid (ATRA; Sigma, Poole, Dorset, UK).

### Plasmid construction and transfection

ADH1-encoding cDNA fragment was cloned into the vector pCMV-N-Flag. Sequences of the clones were confirmed by sequencing. The ADH1 plasmid was transfected by Lipofectamine 3000 (Thermo Fisher Scientific, Waltham, MA, USA) based on information provided by the manufacturer.

### Quantitative real-time PCR (qPCR)

For total RNA preparation, cells were lysed in RNeasy Lysis buffer (Qiagen, Hilden, Germany) containing 1% β-mercaptoethanol. RNA was isolated using the RNeasy RNA preparation microkit (Qiagen, Hilden, Germany) following the instructions provided by the manufacturer. One microgram of total RNA was reverse-transcribed into cDNA using the Superscript II kit (Invitrogen, Carlsbad, CA, USA). The cDNA template was diluted 1:5. The qPCR was carried out using the Bio-Rad Sso Advanced SYBR (172-5261). The PCR data were recorded by Bio-Rad CFX Manager Software (version 2.0). Gene expression data were normalized to the *Gapdh* as house-keeping gene (27). The primers were as follows: *Adh1*, sense, 5′-GCAAAGCTGCGGTGCTATG-3′ antisense, 5′-TCACACAAGTCACCCCTTCTC-3′. *Nr5a1*, sense, 5′-CAGAGCTGCAAAATCGACAA-3′, antisense, 5′-CCCGAATCTGTGCTTTCTTC-3′. *Star*, sense, 5′-gaaAAGACACGGTCATCACTCa-3′, antisense, 5′-CCACCCCTTCAGGTCAATAC-3′. *Cyp11a1*, sense, 5′-AGGTGTAGCTCAGGACTTCA-3′, antisense, 5′-AGGAGGCTATAAAGGACACC-3′. *Hsd3b*, sense, 5′-ACTGCAGGAGGTCAGAGCT-3′, antisense, 5′-GCCAGTAACACACAGAATACC-3′. Cyp17a1, sense, 5′-AGCACCTAGAGGCCGAATCT-3′, antisense, 5′-TGTCTCACCCTTCATTGCTG-3′. *Hsd17b3*, sense, 5′-TCAATGGGACAATGGGCAGT-3′, antisense, 5′-GCTGTGTCATCTTGACTACG-3′. *Gapdh*, sense, 5′-cccACTAACATCAAATGGGG-3′, antisense, 5′-CCTTCCACAATGCCAAAGTT-3′.

### Western blot analysis

Protein extracts from cells and tissues were quantified. Thirty microgram total protein were separated on a 10% SDS-PAGE gel. The separated proteins were transferred to a polyvinylidene difluoride (PVDF) membrane. The membranes were blocked with BSA and incubated at 4°C overnight with primary antibodies anti-ADH1 (CST, Framingham, MA, USA, 1:500), anti-NR5A1 (CST, 1:1,000), anti-HSD3B (Biorbyt, Cambridge, UK, 1:200), anti-StAR (CST, Framingham, MA, USA, 1:1,000), and anti-ALDH1A1 (Protein Tech, Wuhan, China, 1:500). The membranes were washed with tris-buffered saline (TBS buffer), and incubated with horseradish peroxidase (HRP)-conjugated secondary antibodies at room temperature for 1.5 h. The membranes were then washed with TBS buffer three to five times and visualized by enhanced chemiluminescence (ECL) detection. The protein expression was normalized to the GAPDH levels (27).

### Immunofluorescence

For immunostaining, testes were fixed with 4% paraformaldehyde at 4°C overnight, then cryo-embedded in the medium at the optimal cutting temperature (Sakura Finetek, Tokyo, Japan). Sections of 5 μm thickness were blocked with 5% bovine serum albumin (BSA; Sigma) 60 min at room temperature. The primary and secondary antibodies were diluted with 5% BSA. Primary antibodies (CYP17A1, Protein Tech, Wuhan, China) were incubated overnight at 4°C. To visualize nuclei, the sections were stained with DAPI (Thermo Fisher Scientific, Waltham, MA, USA). Sections were photographed under an LSM710 confocal microscope (Zeiss).

### Assay of testosterone concentration

Concentrations of testosterone in the medium and serum were measured with I_125_-testosterone coat-A-count RIA kits (Beijing North Institute of Biological Technology, China). Briefly, the standards, controls, and samples were dispensed into numbered tubes. Subsequently, 100 μl of the I_125_-testosterone tracer and the primary antibody were added to the appropriate tubes. The tubes were shaked and incubated in a water bath for 1 h at 37°C. Then, the secondary antibody was added to all of the tubes, and the mixture was incubated for 15 min at room temperature. The tubes were then centrifuged at 1,800 g for 15 min at 4°C. The supernatants were decanted, and the radioactivity in the precipitate was measured for 1 min. The sensitivity of this assay system was 1 ng/ml. The intra-assay and inter-assay variations were < 10 and 15%, respectively. The results from four separate experiments were averaged for the statistical analysis (28).

### Statistical analysis

Graphpad Prism 6.0 (GraphPad Software, San Diego, CA, USA) was used for statistical analysis. All data were expressed as the mean ± SD. The statistical analyses between two groups was estimated by unpaired two-tailed student's *t*-test. One-way analysis of variance was used for multiple group comparisons. The differences were regarded as significant at *P* < 0.05 (28).

## Results

### Vitamin A-free diet leads to impaired leydig cell differentiation

**Figure 1:**
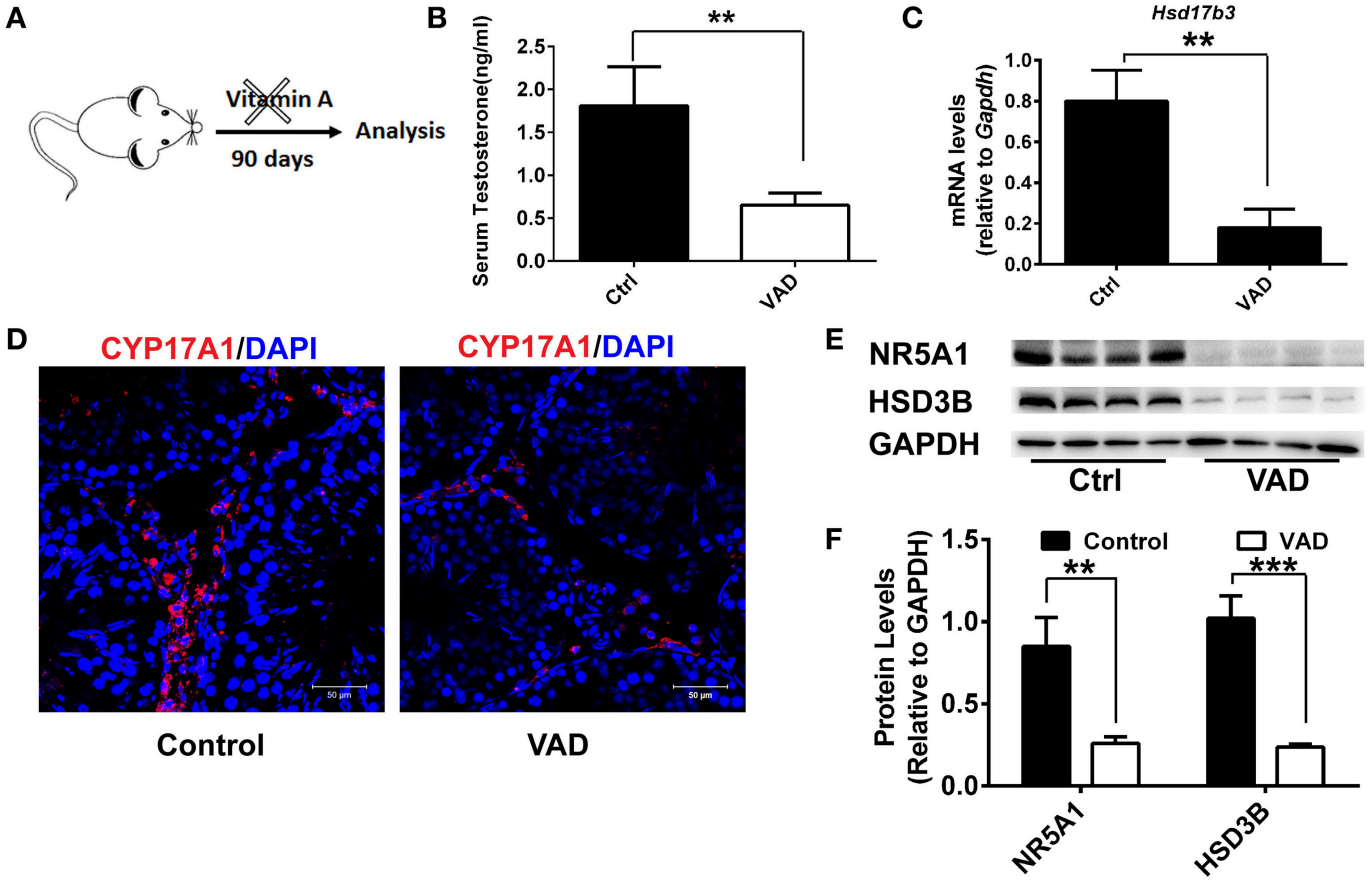
Consequences of a Vitamin A-free diet for the function of Leydig cells. **(A)** Dietary workflow. **(B)** The serum testosterone concentrations of vitamin A-deficient and normal mice were measured by RIA at 90 days. Data are expressed as the mean ± SD of *n* = 8. **(C)** Real-time PCR analysis of the expression levels of *Hsd17b3*, the key gene of testosterone synthesis in the testis of mice with VAD for 90 days. The relative expression was calibrated to *Gapdh*. **(D)** CYP17A1 (red) expression in the testis of mice with VAD for 90 days and normal mice. Nuclei were also stained with DAPI (blue). Scale bar, 50 μm. **(E)** Representative Western blot for protein expression of enzymes (HSD3B and NR5A1) related to steroid synthesis in testis. **(F)** Relative protein expression levels were calibrated to GAPDH. Data are presented as mean ± SD, *n* = 8, ^***^P < 0.001 and ^**^P < 0.01. VAD, vitamin A deficiency, ctrl, control.

To analyze the role of vitamin A in Leydig cell differentiation, we disrupted the RA signaling pathway in mice by a dietary deficiency of vitamin A, which would result in a reduction in levels of endogenous RA. Weanling mice were fed with a vitamin A-free diet for 90 days (vitamin A deficiency, VAD) to prevent the endogenous RA synthesis and deplete the RA reservoir *in vivo* (Figure 1A). Firstly, serum testosterone levels were analyzed after VAD for 90 days. We found the serum testosterone levels had significantly decreased in mice with VAD diet for 90 days compared with the control (Figure 1B). Similarly, the key testosterone synthesis gene *Hsd17b3* also downregulated significantly (Figure 1C). Immunofluorescence was used to analyze the number of Leydig cells. Result suggested the number of Leydig (CYP17A1-positive) cells declined significantly in the interstitium after VAD for 90 days (Figure 1D). To determine the reduced number of Leydig cell was due to deficient differentiation of cell. Key proteins of Leydig cell differentiation, NR5A1 and HSD3B were detected, the results showed the level of NR5A1 and HSD3B significantly decreased in VAD mice (Figures 1E,F). Collectively, these data suggested VAD prevented the synthesis of RA, which depressed testosterone production and expression of the key protein of Leydig cell differentiation. VAD adversely affected the differentiation and testosterone production of Leydig cells.

### Metabolic enzymes involved in vitamin A are associated with leydig cell differentiation

**Figure 2:**
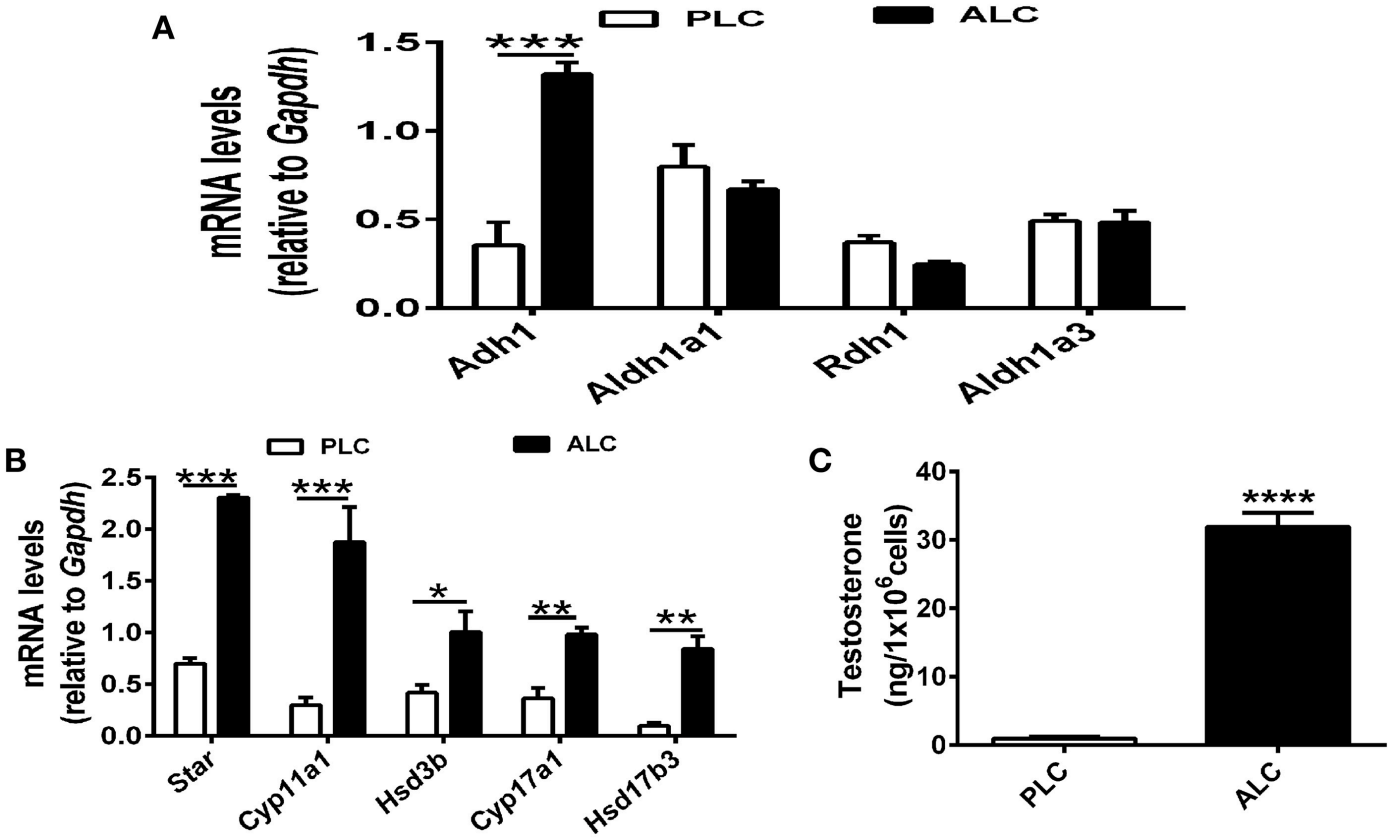
ADH1 correlates with Leydig cell differentiation. **(A)** The levels of mRNA related to RA synthesis in progenitor Leydig cells and adult Leydig cells. **(B)** Analysis of the expression of key genes encoding steroidogenic enzymes in progenitor Leydig cells and adult Leydig cells. **(C)** Testosterone production in progenitor Leydig cells and adult Leydig cells. Cells were cultured in Leydig cell medium for 24 h, and the hormone secreted into the medium were measured by RIA. PLC, progenitor Leydig cell. ALC, adult Leydig cell. All quantitative data were obtained from three independent experiments and are presented as mean ± SD; ^****^P < 0.0001, ^***^P < 0.001, ^**^P < 0.01, and ^*^P < 0.05.

RA (biologically active metabolite of vitamin A) and is produced in two steps from dietary vitamin A by different enzymes, there are discrepancies regarding which cell types express particular enzymes. To determine the mechanism of vitamin A in Leydig cell differentiation, we analyzed the expression of *Adh1, Aldh1a1, Rdh1*, and *Aldh1a3* in progenitor Leydig cells and adult Leydig cells. We observed that *Adh1* exhibited significant changes in expression between progenitor Leydig cells and adult Leydig cells, whereas the expression of *Aldh1a1, Rdh1*, and *Aldh1a3* in progenitor Leydig cells did not show significant change when compared with adult Leydig cells (Figure 2A). Additionally, we analyzed the change of Leydig markers. The well-established Leydig cell markers (*Star, Cyp11a1, Hsd3b*, and *Cyp17a1*) were upregulated in adult Leydig cells compared with progenitor Leydig cells (Figure 2B). Moreover, we also examined the testosterone level in adult Leydig cells and progenitor Leydig cells. The data suggested that adult Leydig cells testosterone production was increased significantly when compared with progenitor Leydig cells (Figure 2C). These results demonstrated that *Adh1* positively correlated with the expression of Leydig cell steroidogenic genes and testosterone synthesis, which raised an interesting question on whether RA is involved in the Leydig cell differentiation via *Adh1*.

### ADH1 regulates leydig cell differentiation *in vitro*

According to our findings above, ADH1 showed a positive correlation with the differentiation of Leydig cells. To illustrate whether ADH1 contribute to leydig cells differentiation. We studied the role of ADH1 in the context that directed stem cells differentiating to functional Leydig cells. Since stem Leydig cells only exist in fetal mice and are few in number, it is very difficult to separate them. In our previous study we have explored an induced functional Leydig cell, mESCs-SF1, which was derived from mouse embryonic stem cell and overexpressing SF1. This transgene ESCs could be differentiated into Leydig cell and produce testosterone after being treated with Leydig cell differentiation medium (LC DM) for 6 days (27). Using these induced Leydig cells as a model, we could accurately assess whether altered ADH1 level could impact Leydig cell differentiation.

**Figure 3:**
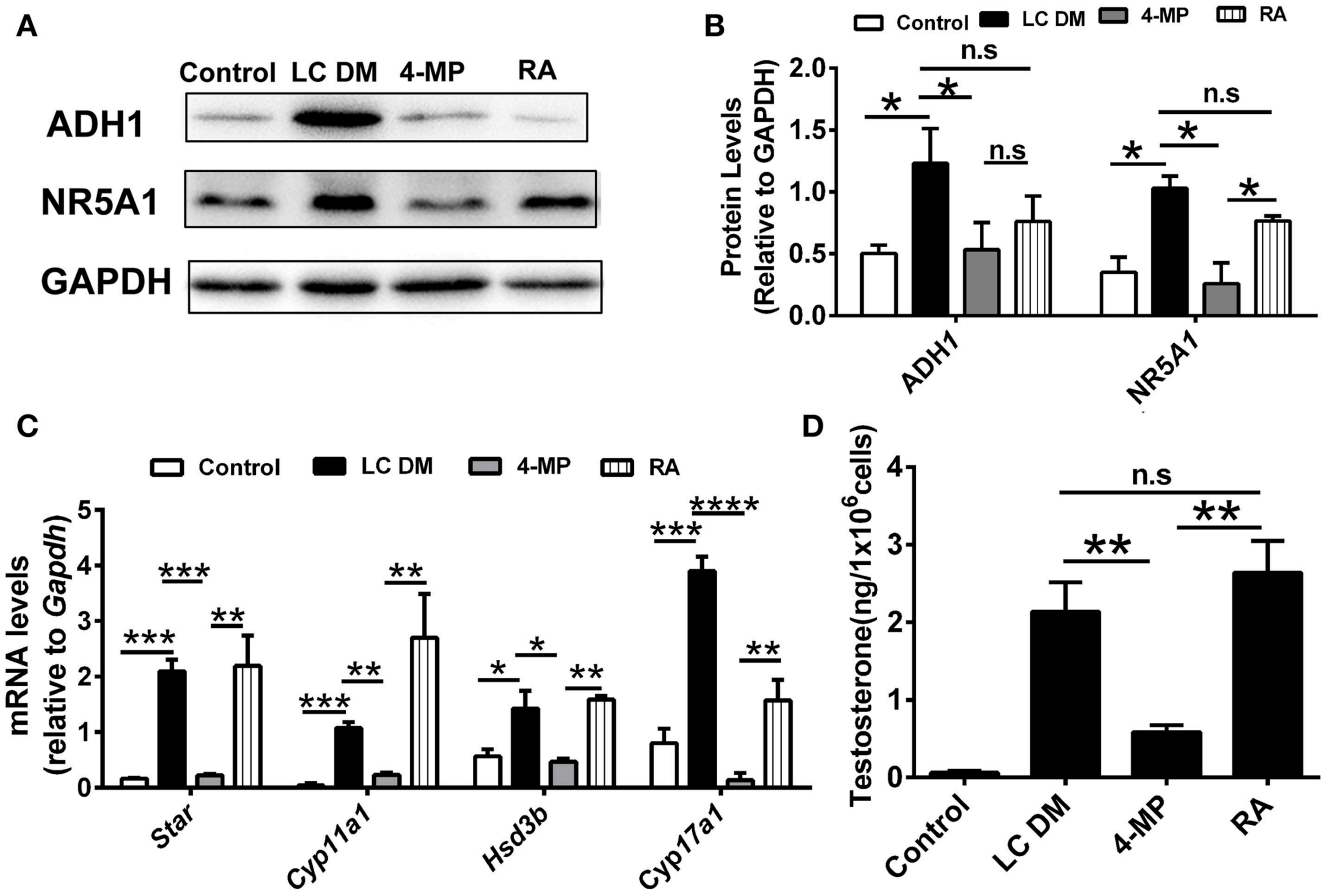
Inhibition of ADH1 represses Leydig cell differentiation *in vitro*. **(A,B)** Representative Western blot for protein expression of ADH1, and NR5A1 in different culture conditions at day 6. Relative protein expression levels were normalized to GAPDH. **(C)** Analysis of the expression of key genes involved in steroidogenic enzymes under different culture conditions using real-time PCR. **(D)** Testosterone production in different culture conditions at day 6. LC DM, Leydig cell differentiation medium. 4-MP, LC DM with the ADH1 inhibitor 4-MP, RA, LC DM medium with the 4-MP and RA. All quantitative data were obtained from three independent experiments and are presented as mean ± SD; ^****^P < 0.0001, ^***^P < 0.001, ^**^P < 0.01, and ^*^P < 0.05.

**Figure 4:**
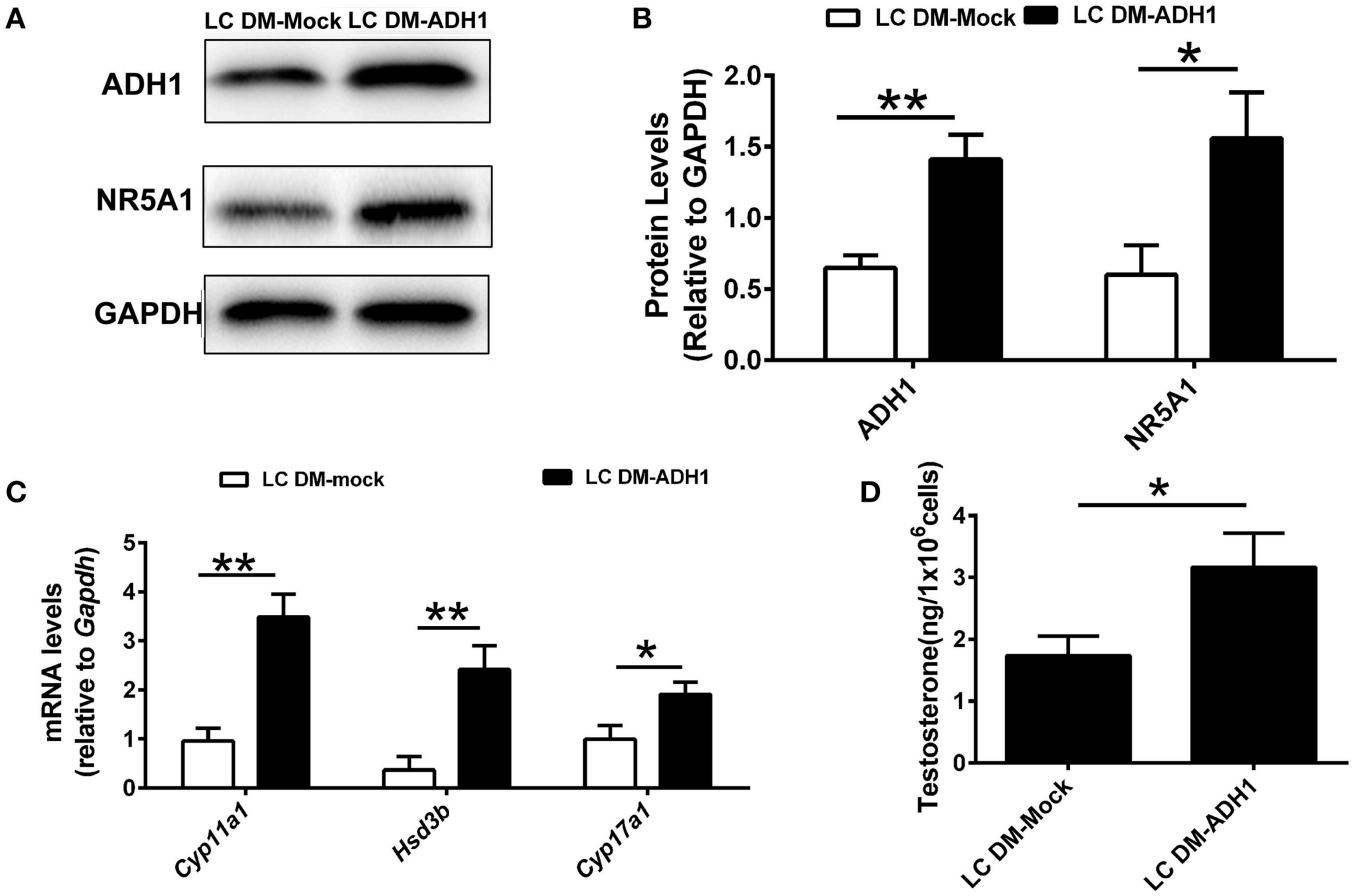
Overexpression of ADH1 promotes mESC differentiation into Leydig cells. **(A,B)** Representative Western blot for protein expression of ADH1 and NR5A1 in SF1-overexpressing mESCs (mESCs-SF1) transfected with adenovirus (empty vector or *Adh1*) at day 6. Relative protein expression levels were normalized to GAPDH. **(C)** Analysis of the expression of key genes involved in steroidogenic enzymes in mESCs-SF1 transfected with empty vector or *Adh1*. **(D)** Testosterone production in different culture conditions at day 6. All quantitative data were obtained from three independent experiments and are presented as mean ± SD; ^**^P < 0.01 and ^*^P < 0.05. LC DM-Mock, mESCs-SF1 transfected with empty vector, then cultured in Leydig cell differentiation medium (LC DM) for 6 days. LC DM-ADH1, mESCs-SF1 transfected with *Adh1*, then cultured in LC DM for 6 days.

The ADH1 inhibitor 4-MP was supplied to the Leydig cells differentiation medium. ADH1 expression was downregulated by 4-MP. The expression of Nr5a1 also decreased significantly upon 4-MP treatment (Figures 3A,B). Then, the effects of 4-MP on expression levels of steroidogenesis-related genes (*Star, Cyp11a1, Hsd3b*, and *Cyp17a1*) in Leydig cells were analyzed. The results showed that the level of steroidogenic genes was significantly inhibited by 4-MP (Figure 3C). Similarly, the capability to produce testosterone was also greatly repressed by 4-MP (Figure 3D). These data demonstrate that alteration ADH1 levels could impact Leydig cell differentiation. As the synthesis of RA depends on ADH1 in Leydig cells, the 4-MP could decrease the synthesis of RA. Therefore, we also tested whether treatment with the RA signaling agonist all-trans retinoic acid (ATRA) could also contribute to the SF1-overexpressing mESCs differentiating to Leydig cells. As expected, the ATRA could reverse the suppression of 4-MP. ATRA significantly stimulated the expression of steroidogenic genes (*Nr5a1, Cyp11a1, Hsd3b, Cyp17a1*), the NR5A1 protein, and testosterone production (Figure 3). On the contrary, when overexpressed *Adh1*, the expression of NR5A1 protein and steroidogenic genes (*Cyp11a1, Hsd3b, Cyp17a1*) were significantly upregulated compared to control (Figures 4A–C). More important, overexpression of *Adh1* could promote testosterone production significantly (Figure 4D). Based on these data, we could conclude that ADH1 was involved in regulating Leydig cell differentiation and function by regulating RA synthesis.

**Figure 5:**
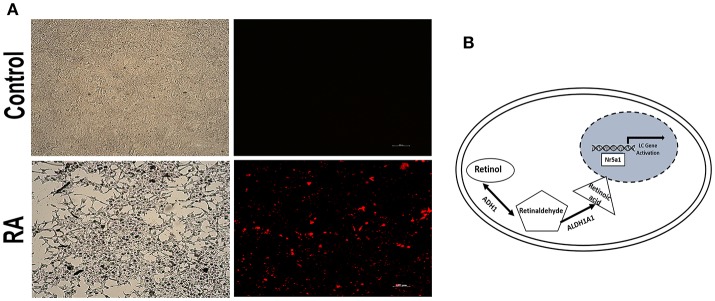
RA activated the Nr5a1 promoter activity. **(A)** Fluorescence was visualized by microscope after cells transiently transfected with a vector carrying the mCherry gene driven by the *Nr5a1* promoter (−1134 to +19) or empty vector and treated with all-trans RA for 24h. Scale bar 100 μm. **(B)** Schematic representation of the mechanism of action of ADH1 in Leydig cell differentiation. Upregulation of ADH1 stimulated RA synthesis, and subsequently, RA facilitated Leydig cell differentiation by activating the *Nr5a1* promoter, which activated the expression of specific genes (*Star, Hsd3b, Cyp11a1*, and *Cyp17a1*) resulting in precursor Leydig cell differentiation into the functional Leydig cell.

Additionally, to assess whether RA affects the Nr5a1 promoter activity, Leydig cells were transiently transfected with the Nr5a1 promoter (−1134 to +19) driven mCherry reporter gene plasmid. Then the cells were treated with ATRA. The results showed that RA significantly activates the Nr5a1 promoter activity (Figure 5A). In summary, these data revealed a novel role for ADH1, the metabolic enzyme involved in vitamin A, as a regulator of Leydig cell differentiation *in vitro*. Upregulation of ADH1 could increase the RA reservoir and RA could then facilitate Leydig cell differentiation by activating the *Nr5a1* promoter activity (Figure 5B).

### ADH1 regulates leydig cell differentiation *in vivo*

**Figure 6:**
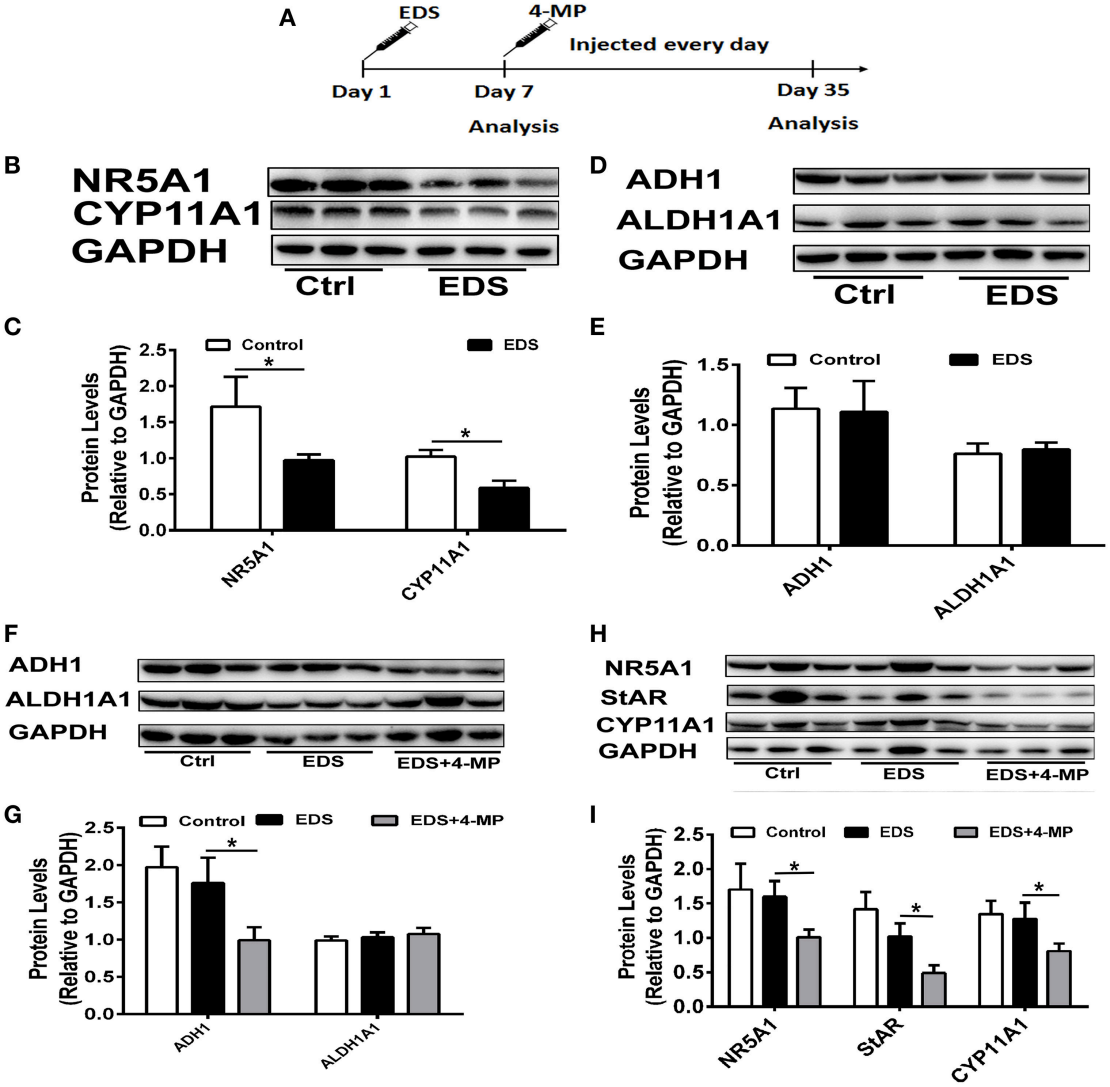
Inhibition of ADH1 level repress Leydig cell differentiation *in vivo*. **(A)** Schematic presentation of the experimental design. **(B,C)** The expression of the NR5A1 and CYP11A1 in Leydig cells at 7 days after EDS treatment. **(D,E)** The expression of enzymes ADH1 and ALDH1A2, which are involved in RA synthesis, at 7 days after EDS treatment. **(F,G)** The effects of 4-MP on the expression of the enzymes ADH1 and ALDH1A2 at 35 days after EDS treatment. **(H,I)** The effects of 4-MP on the expression of the NR5A1 and CYP11A1 at 35 days after EDS treatment. Relative protein expression levels were normalized to GAPDH. Data are expressed as the mean ± SD of *n* = 8, ^*^P < 0.05.

To further identify the role of ADH1 in Leydig cell differentiation, the effect of altered ADH1 levels on Leydig cell differentiation was examined *in vivo* (Figure 6A). Previous studies have proved that EDS could selectively induce apoptosis of mature Leydig cells, and mesenchymal-like precursor Leydig cells start to proliferate and further differentiate into mature Leydig cells after EDS treatment (29). This is an ideal model for studying the differentiation of Leyidg cell *in vivo*. Because it has been reported that regeneration of identifiable new adult Leydig cells occur from 14 days post-EDS, with recovery to normal adult Leydig cell numbers by week 5 after complete adult the Leydig cells ablation by EDS (30). Consistent with our earlier experiment results, expression of steroidogenic enzyme such as CYP11A1 and NR5A1 decreased significantly upon EDS treatment (Figures 6B,C). However, the expression levels of ADH1 and ALDH1A1 did not show any change after EDS treatment (Figures 6D,E).

Then we applied 4-MP at day 7 after EDS treatment to examine whether 4-MP would affect Leydig precursor cell differentiation to functional Leydig cells. To exclude possible artifacts caused by 4-MP, the model was only solvent injection. We analyzed the expression of steroidogenic proteins and serum testosterone levels at week 5 after EDS injection. As expected, injection of the ADH1 inhibitor 4-MP resulted in significant decreasing ADH1 expression, while no obvious change in ALDH1A1 expression. (Figures 6F,G). These results suggested that 4-MP could also specifically inhibit ADH1 expression *in vivo*. The expression of NR5A1, StAR, and CYP11A1 was significantly higher compared with the 4-MP treatment group, and there was no significant change between the EDS group and the control (Figures 6H,I). Together, these results demonstrated that decreasing ADH1 level inhibited the differentiation of Leydig cell *in vivo*.

## Discussion

Leydig cell differentiation is a complex process regulated by many signals from the testis environment. Here we identified that vitamin A is involved in the Leydig cells differentiation by RA. ADH1 plays an essential role in controlling the conversion of vitamin A to RA, whereas the other metabolic enzymes involved in vitamin A did not change when progenitor Leydig cells differentiated to adult Leydig cells. Upregulation of ADH1 stimulated RA synthesis, and subsequently, RA facilitated Leydig cell differentiation by activating the *Nr5a1* promoter, which upregulated the expression of specific genes (*Star, Hsd3b, Cyp11a1*, and *Cyp17a1*) resulting in precursor Leydig cell differentiation into the functional Leydig cell. Our study connected a metabolic enzyme of RA signaling to the regulation of Leydig cell differentiation.

The regulation of gene expression by vitamin A and its bioactive metabolites is a well-characterized example of direct nutrient regulation of gene expression (31). Previous studies have demonstrated that the vitamin A plays a role in testis development (32, 33); vitamin A can promote Sertoli cell (34, 35) and germ cell (35, 36) differentiation. However, little is known about vitamin A in the differentiation of Leydig cells. In our study, we disrupted retinoic acid signaling pathway in mice by a dietary deficiency of vitamin A (vitamin A-free diet for 90 days), then testes were analyzed. The results suggested that serum testosterone significantly decreased in VAD mice. Additionally, the number of CYP17A1 positive Leydig cell declined significantly in the interstitium after initial deficiency. To determine the reduced number of Leydig cell was due to deficient differentiation of cell. Key proteins of Leydig cell differentiation, NR5A1 and HSD3B were analyzed, the results demonstrated the expression of NR5A1 and HSD3B significantly decreased in VAD mice. These results suggested that vitamin A was necessary for maintaining the differentiation of Leydig cells *in vivo*.

To illustrate the mechanism of vitamin A in differentiation of Leydig cell, we analyzed the expression of *Adh1, Aldh1a1, Rdh1*, and *Aldh1a3* in precursor Leydig cells and adult Leydig cells, of which only ADH1 was upregulated. ADH1 is an important regulator of Leydig cell differentiation. Altering ADH1 level could affect Leydig cell differentiation by overexpressing ADH1 gene and ADH1 inhibitor *in vitro* cell experiments. And ADH1 exerted its effects on Leydig cell differentiation by controlling the conversion of vitamin A to RA, which in turn affected NR5A1 expression and upregulated the level of specific genes. And this conclusion was further confirmed by *in vivo* experiments.

NR5A1 is known to contribute to Leydig cell differentiation (37). Mutations in the human SF1 gene are also a frequent cause of 46 XY disorders of sex development and are often associated with improper Leydig cell differentiation and function (38–40). NR5A1 is sufficient to induce stem cells differentiating into steroidogenically active Leydig cells (24–27, 41). In our study, the vitamin A metabolizing enzyme ADH1 promoted NR5A1 expression by increasing RA levels. However, the molecular mechanisms of vitamin A in the regulation of NR5A1 require further investigation.

Nevertheless, our study reveals that vitamin A regulates Leydig cell differentiation, ADH1 serves as an important factor involved in the conversion of vitamin A to RA. Upregulation of ADH1 can lead to an increase in RA levels, which consequently increases the expression of Nr5a1, resulting in Leydig cell differentiation. The connecting between molecular biology and nutrition provides new insights into the mechanisms by which nutrients directly regulate the differentiation of Leydig cell. A better mechanistic understanding of the differentiation effects exerted by ADH1, which also provide experimental evidence for the development of therapeutics to promote Leydig regeneration through the administration of a RA signaling regulator or a vitamin A supplement.

## Author contributions

YY, JL, DY, TZ, QiL, XW, and QuL performed experiments. ZS, QZ and QX analyzed data. YY and YH designed experiments and wrote the manuscript.

### Conflict of interest statement

The authors declare that the research was conducted in the absence of any commercial or financial relationships that could be construed as a potential conflict of interest.
